# Homofermentative production of optically pure L-lactic acid from xylose by genetically engineered *Escherichia coli* B

**DOI:** 10.1186/1475-2859-12-57

**Published:** 2013-06-07

**Authors:** Jinfang Zhao, Liyuan Xu, Yongze Wang, Xiao Zhao, Jinhua Wang, Erin Garza, Ryan Manow, Shengde Zhou

**Affiliations:** 1 Hubei Provincial Cooperative Innovation Center of Industrial Fermentation, Key Laboratory of Fermentation Engineering (Ministry of Education), College of Bioengineering, Hubei University of Technology, Wuhan 430068, PR China; 2 Department of Biological Sciences, Northern Illinois University, DeKalb, IL, 60115, USA

**Keywords:** *E. coli*, Genetic engineering, L-lactic acid, PLA, Xylose fermentation

## Abstract

**Background:**

Polylactic acid (PLA), a biodegradable polymer, has the potential to replace (at least partially) traditional petroleum-based plastics, minimizing “white pollution”. However, cost-effective production of optically pure L-lactic acid is needed to achieve the full potential of PLA. Currently, starch-based glucose is used for L-lactic acid fermentation by lactic acid bacteria. Due to its competition with food resources, an alternative non-food substrate such as cellulosic biomass is needed for L-lactic acid fermentation. Nevertheless, the substrate (sugar stream) derived from cellulosic biomass contains significant amounts of xylose, which is unfermentable by most lactic acid bacteria. However, the microorganisms that do ferment xylose usually carry out heterolactic acid fermentation. As a result, an alternative strain should be developed for homofermentative production of optically pure L-lactic acid using cellulosic biomass.

**Results:**

In this study, an ethanologenic *Escherichia coli* strain, SZ470 (Δ*frdBC* Δ*ldhA* Δ*ackA* Δ*pflB* Δ*pdhR ::pflBp6-acEF-lpd* Δ*mgsA)*, was reengineered for homofermentative production of L-lactic acid from xylose (1.2 mole xylose = > 2 mole L-lactic acid), by deleting the alcohol dehydrogenase gene (*adhE*) and integrating the L-lactate dehydrogenase gene (*ldhL*) of *Pediococcus acidilactici*. The resulting strain, WL203, was metabolically evolved further through serial transfers in screw-cap tubes containing xylose, resulting in the strain WL204 with improved anaerobic cell growth. When tested in 70 g L^-1^ xylose fermentation (complex medium), WL204 produced 62 g L^-1^ L-lactic acid, with a maximum production rate of 1.631 g L^-1^ h^-1^ and a yield of 97% based on xylose metabolized. HPLC analysis using a chiral column showed that an L-lactic acid optical purity of 99.5% was achieved by WL204.

**Conclusions:**

These results demonstrated that WL204 has the potential for homofermentative production of L-lactic acid using cellulosic biomass derived substrates, which contain a significant amount of xylose.

## Background

Lactic acid, a widely used chemical, exists as a mixture of D and L isomers when synthesized through a chemical route [1]. The requirement of an optically pure L isomer for applications in pharmaceutical and poly-lactic acid (PLA) bioplastic industries favors fermentative production of L-lactic acid using chiral-specific L-lactate dehydrogenase.

Glucose derived from starch biomass such as corn, is currently used for fermentative production of L-lactic acid by lactic acid bacteria like *Lactobacillus*. However, due to its competition with food resources, an alternative non-food substrate is needed for cost-effective production of L-lactic acid, in order to enable the environmentally friendly PLA to compete economically with petroleum based plastics [2].

Cellulosic biomass, the most abundant non-food resource, is a potential substrate for L-lactic acid fermentation. However, in addition to glucose, the substrate (sugar stream) derived from cellulosic biomass contains significant amounts of xylose, which is unfermentable by most lactic acid bacteria [2]. The microorganisms that do ferment xylose to L-lactic acid, such as *Lactococcus lactis* IO-1 [3] and *Enterococcus mundtii*[4], need improvements in yield, productivity, optical purity, and/or the requirement of complex nutrients.

*Escherichia coli*, a candidate with minimal nutrient requirements*,* is able to use all biomass derived hexose and pentose sugars. Derivative *E. coli* strains have been engineered for production of lactic acid [5-12]. However, few of these *E. coli* strains have demonstrated the ability to ferment xylose into L-lactic acid at high yields and/or optical purity. Furthermore, *E. coli* naturally produces D-lactic acid and lacks an endogenous L-lactate dehydrogenase gene. A plasmid bearing an exogenous L-lactate dehydrogenase gene from *Streptococcus bovis*[6,13], *Lactobacillus casei*[14], or *Clostridium thermocellum*[9] has been cloned into *E. coli* (*pfl ldhA*) to produce L-lactic acid. These plasmid bearing recombinants, however, may lack strain stability due to plasmid curing.

In this study, we report reengineering an ethanologenic *E. coli* strain, SZ470 (*ΔfrdBC ΔldhA ΔackA ΔfocA-pflB ΔpdhR::pflBp6-pflBrbs-aceEF-lpd*) [15], for homofermentative production of L-lactic acid from xylose. The resulting strain, WL204, contains a chromosomal integrated *ldhL* gene, without any antibiotic marker or plasmids.

## Results and discussion

### Eliminate ethanol pathway

*E. coli* SZ470 (Δ*frdBC* Δ*ldhA* Δ*ackA* Δ*pflB* Δ*pdhR::pflBp6-acEF-lpd* Δ*mgsA*), a xylose fermenting homoethanol strain previously engineered from *E. coli* B, was selected for reengineering to produce L-lactic acid using xylose. The ethanol pathway of SZ470 was eliminated through deletion of the endogenous alcohol dehydrogenase gene (*adhE*) using the *adhE*’-FRT-*kan*-FRT-*adhE’* DNA fragment and the λ red recombinase system [10,16]. The antibiotic marker (*kan*) of the resulting kanamycin resistant colonies was then removed from the chromosome through FRT-recognizing site specific recombinase (flipase), producing the strain WL202 (∆*adhE*::FRT) which lacks the ethanol pathway and antibiotic marker.

**Figure 1:**
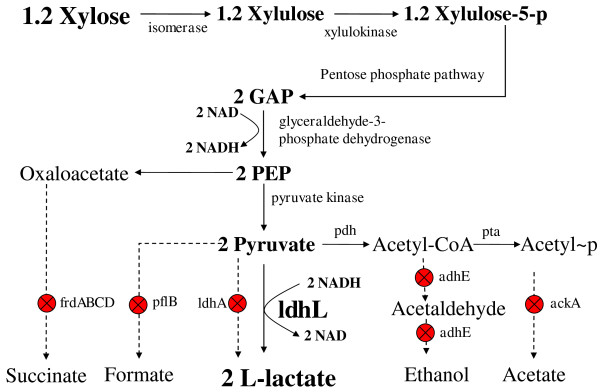
**Engineering a xylose-to-L-lactic acid homofermentative pathway (1.2 mole xylose = > 2 mole L-lactic acid).** Genes encoding important enzymes are indicated by italics. The relevant gene encoding enzymes are: *pdh*, pyruvate dehydrogenase complex (engineered for anaerobic expression in a previous study); *pta*, phosphotransacetylase; *adhE*, alcohol dehydrogenase; *ackA*, acetate kinase; *ldhA*, D-lactate dehydrogenase; *ldhL*, L-lactate dehydrogenase (expressed from *ldhA* promoter); *pflB*, pyruvate formate lyase; *frdABCD*, fumurate reductase. The abbreviated metabolic intermediates are: GAP, glyceraldehyde-3-phosphate; PEP, phosphoenol pyruvate. The stop sign indicated the relevant gene was deleted.

Fermentation tests showed that WL202 lost its ability to produce ethanol and anaerobic cell growth, indicating *adhE* was successfully deleted. The loss of anaerobic growth was expected since the only NADH oxidation pathway (through the ethanol pathway) of WL202 was blocked by deletion of *adhE* (Figure 1).

### Establish lactate pathway

To regain anaerobic growth and the ability to produce L-lactic acid by WL202, a L-(+)-lactate dehydrogenase enzyme was needed to convert pyruvate to L-lactate and oxidize NADH, and establish a homolactate pathway with a balanced NADH/NAD redox (1 glucose or 1.2 xylose + 2 NAD (glycolysis) = > 2 pyruvate + 2 NADH (L-lactate dehydrogenase) = > 2 L-lactate + 2 NAD) (Figure 1). To this end, the L-(+)-lactate dehydrogenase gene (*ldhL*) of *Pediococcus acidilactici* was amplified by PCR using *E. coli* SZ85 chromosomal DNA as the template (a strain containing integrated *ldhL*) [17]. The amplified DNA fragment contained the *ldhL* coding region flanked by the promoter and terminator of the native *E. coli ldhA* gene. This hybrid DNA fragment (*ldhA* promoter-*ldhL* -*ldhA* terminator) was then transformed into WL202 (pKD46) through electroporation. The double homologous recombination with *ldhL* integrated at the *ldhA* locus was selected through anaerobic cell growth in screw-cap tubes. The temperature sensitive plasmid, pKD46, was then cured by incubation at 42°C. The resulting strain was designated WL203 (∆*ldhA*::*ldhL*).

WL203 produced L-lactic acid from xylose in screw cap tubes and in small scale fermentation with limited anaerobic growth (OD_600nm_ of 0.5-1.0 after 24 h). A growth based metabolic evolution process was then carried out by growing and serial transferring (at 24 h intervals) WL203 in a LB-xylose medium for three months, resulting in strain WL204 with a one-fold improvement in anaerobic cell growth in screw cap tubes.

### L-lactic acid production from xylose

**Figure 2:**
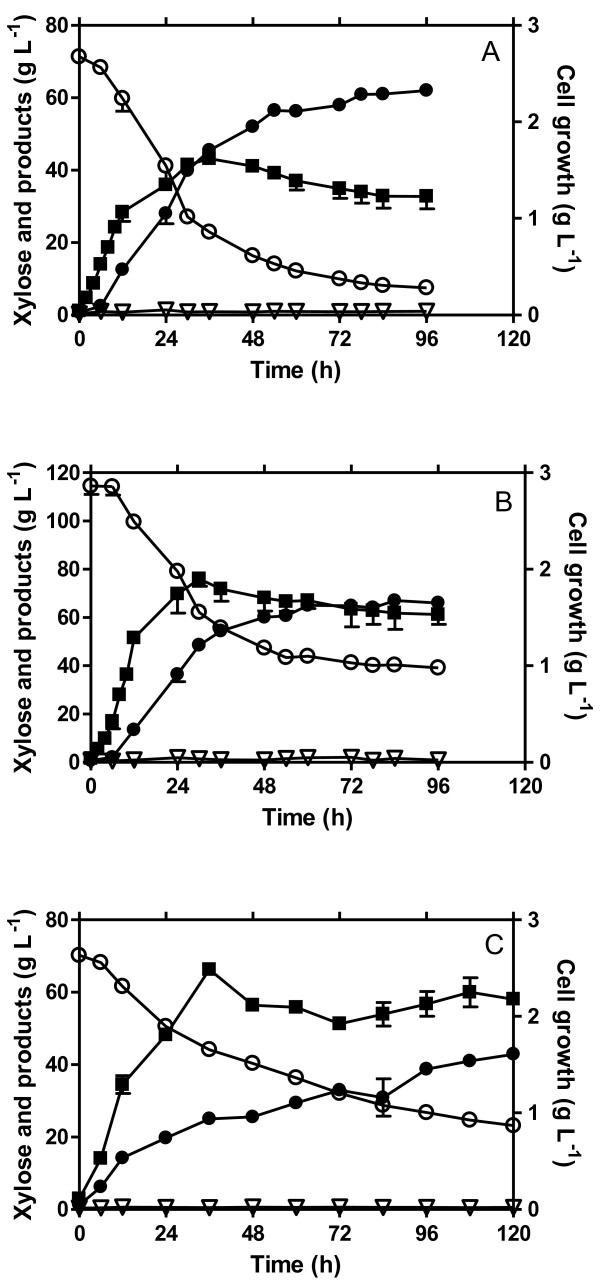
**L-lactic acid fermentation from xylose. A**) 70 g L^-1^ xylose LB medium; **B**) 100 g L^-1^ xylose LB medium; **C**) 70 g L^-1^ xylose NBS medium. Symbols: open circle, xylose; filled circle, lactic acid; filled square, cell growth; triangle, acetic acid. Each data point is the average of three replicates with the error bar representing the standard deviation.

L-lactic acid production by *E. coli* WL204 was evaluated using a 7-L fermenter containing 70 g L^-1^ xylose (pH 7.0, 37°C). The results are shown in Figure 2A and Table 1. During the initial 12 h fermentation, WL204 achieved the maximum growth rate of 0.271 h^-1^, and then continued to grow at a lower rate for another 24 h, reaching the maximum biomass of 1.619 g L^-1^. There was little lactic acid produced for the first 6 h while the cell was actively growing up to one third of the maximum biomass. Nevertheless, 39 g L^-1^ lactic acid was produced in the following 24 h production period (6 h-30 h), maintaining the maximum production rate of 1.631 g L^-1^. Lactic acid was continuously produced at a lower rate for the next 48 h (36 h-78 h), achieving an average production rate of ~0.78 g L^-1^ and a final titer of 62 g L^-1^for the active production period (0 h-78 h). Little lactic acid, if any, was produced thereafter even though there was 7.48 g L^-1^ xylose still available. Nevertheless, a 97% theoretical yield was achieved based on the xylose metabolized, indicating that xylose was converted to lactic acid at a nearly perfect 1:1.667 molar ratio, with little acetic acid (~1 g L^-1^) produced as a by-product.

**Table 1:** **Summary of xylose fermentations by *E. coli *WL204**

**Xylose (g L**^**-1**^**)**	**Cell growth**		**Lactic acid**		**Volumetric productivity (g L**^**-1**^**h**^**-1**^**)**		**Specific productivity (g g**^**-1**^**h**^**-1**^**)**		**By-product (Acetic acid) (g L**^**-1**^**)**
	**Mass (g L**^**-1**^**)**	**Rate**^**1) **^**(h**^**-1**^**)**	**Titer (g L**^**-1**^**)**	**Yield**^**2) **^**(%)**	**Maximum**^**3)**^	**Average**^**4)**^	**Maximum**^**3)**^	**Average**^**4)**^	
70 (LB)	1.619 ± 0.112	0.271 ± 0.032	62.04 ± 0.92	97	1.631 ± 0.039	0.780 ± 0.017	1.05 ± 0.032	0.623 ± 0.097	1.01 ± 0.383
100 (LB)	1.901 ± 0.110	0.291 ± 0.030	66.03 ± 1.51	90	1.934 ± 0.011	1.092 ± 0.010	1.02 ± 0.054	0.653 ± 0.052	0.992 ± 0.087
70 (NBS)	2.487 ± 0.150	0.205 ± 0.020	42.9 ± 0.80	91	0.768 ± 0.021	0.358 ± 0.012	0.309 ± 0.018	0.144 ± 0.010	0.596 ± 0.026

^1)^ The growth rate was calculated from the log growth period (0 h to 12 h).

^2)^ The yield was calculated based on lactic acid produced over xylose metabolized.

^3)^ The maximum productivity was calculated from the fastest 24 h fermentation period (6 h to 30 h for both 70 g L^-1^ and 100 g L^-1^ xylose LB medium; 0 h to 24 h for 70 g L^-1^ xylose NBS medium).

^4)^ The average productivity was calculated from the active production period (0 h to 78 h for 70 g L^-1^ xylose fermentation; 0 h to 60 h for 100 g L^-1^xylose fermentation, 0 h to 120 h for 70 g L^-1^ xylose NBS fermentation).

100 g L^-1^ xylose fermentation was also carried out to evaluate if osmotic pressure (from xylose) would be a challenge for cell growth and lactic acid production by WL204. As the results show in Figure 2B and Table 1, the patterns of cell growth, xylose consumption and lactic acid production were similar to those of the 70 g L^-1^ xylose fermentation. The cell growth rate (0.291 h^-1^), biomass (1.901 g L^-1^), and the maximum (1.934 g L^-1^h^-1^) and average (1.092 g L^-1^h^-1^) volumetric productivities of 100 g L^-1^ xylose fermentation were 7.4%, 17.4%, 18.6%, and 40% higher, respectively, than those of the 70 g L^-1^ xylose fermentation, demonstrating that osmotic pressure from 100 g L^-1^ xylose presented little challenge for WL204. However, the 66 g L^-1^ lactic acid titer achieved was almost the same as that achieved (62 g L^-1^) in the 70 g L^-1^ xylose fermentation, indicating that 62-66 g L^-1^ lactate inhibited WL204 from further growth and fermentation.

WL 204 was further evaluated for its ability for L(+)-lactic acid production in mineral salts medium using 70 g L^-1^ xylose NBS fermentation. As demonstrated in Figure 2C and Table 1, the engineered WL204 maintained its ability to grow and produce lactic acid with over 90% product yield in mineral salts medium. However, cell growth rate, product titer, and productivity achieved in mineral salts medium (NBS) were 32%, 44%, and 117% lower, respectively, than those obtained in complex LB medium under the same condition, suggestion that WL204 needs further adaptive evolution to improve its growth rate and lactic acid production in NBS medium.

### Optical purity of L-lactic acid

Only the L-lactic acid isomer was detected in the fermentation product when analyzed by HPLC using a chiral column. However, a D-isomer peak was observed if it was intentionally added into the fermentation product at a ratio of 0.5% of the total lactic acid. These results indicated that the optical purity of L-lactic acid produced was at least 99.5%.

## Conclusions

The current biocatalysts used for commercial L-lactic acid production are either unable to metabolize xylose or they metabolize xylose through phosphoketolase, which leads to a heterofermentative pathway of equal molar lactic acid and acetic acid [18]. Attempting to engineer homolactic acid production from xylose has met limited success in lactic acid bacteria [18,19] and in *E. coli* strains [6,14,17].

By deleting the alcohol dehydrogenase gene (*adhE*) of a previously engineered xylose fermenting ethanologenic *E. coli* strain, and integrating an *ldhL* gene into the chromosome, we successfully engineered an L-lactic acid producing strain, WL204, which produced 62-66 g L^-1^ L-lactic acid, has a productivity of 1.631-1.934 g L^-1^, a yield of 90-97%, and an optical purity of 99.5% from xylose fermentation (complex medium). These results are favorable when compared to those achieved by the prior reported *E. coli* strains for L-lactic acid fermentation from xylose (Table 2). Nevertheless, further improvement of lactic acid tolerance, growth rate and productivity in mineral salts medium of WL204 is needed to achieve a cost effective titer (≥ 100 g L^-1^) for practical applications.

**Table 2:** **Comparison of *E. coli *strains engineered for L-lactic acid production using xylose**

**Strains**	***ldhL *location**	**Medium**	**Initial xylose (g L**^**-1**^**)**	**L-lactic acid**				**Reference**
				**Titer (g L**^**-1**^**)**	**Productivity (g L**^**-1**^**h**^**-1**^**)**	**Yield (%)**	**Optical purity (%)**	
*E. coli* FBR9	plasmid	complex	100	56	0.47	84	n/a	[14]
*E. coli* FBR11	plasmid	complex	100	63	0.73	89	n/a	[14]
*E. coli* FBR19	plasmid	complex	40	32	n/a	88	n/a	[6]
*E. coli* SZ85	chromosome	minimal	50	40	0.32	93	99.5	[17]
*E. coli* WL204	chromosome	complex	100	66	1.09	90	99.5	This study

## Methods

### Strains, plasmids and growth conditions

The bacterial strains, plasmids, and primers used in this study are listed in Table 3. Bacterial cultures were grown at 37°C in Luria–Bertani (LB) broth (g L^-1^: tryptone 10, yeast extract 5, and NaCl 5) supplemented with 20 g L^-1^ xylose, or on LB plates (agar 20 g L^-1^) containing 20 g L^-1^ xylose. 50 μg ml^-1^ kanamycin or ampicillin was added into the medium as needed during strain construction [10]. Fermentations were carried out in both complex LB medium and mineral salts medium (NBS medium (g L^-1^): KH_2_PO_4_, 3.5; K_2_HPO_4_, 5.0; (NH_4_)_2_HPO_4_, 3.5; MgSO_4_:7H_2_O, 0.25; CaCl_2_:2H_2_O, 0.015; thiamine, 0.0005; and 1 mL of trace metal stock). The trace metal stock was prepared as previously described [10].

**Table 3:** ***E. coli *strains, plasmids and primers used in this study**

**Strains**	**Relevant characteristics**	**Sources**
SZ470	*E. coli* B, Δ*frdBC* Δ*ldhA* Δ*ackA* Δ*pflB* Δ*pdhR ::pflBp6-acEF-lpd* Δ*mgsA*	[15]
SZ85	*E. coli* W3110, △*focA-pflB* △*frdBC* △*adhE* △*ackA* △*ldhA*::*ldhL*	[17]
WL202	SZ470, △*adhE*, lost anaerobic growth	This study
WL203	WL202, △*ldhA*::*ldhL*, regained anaerobic growth	This study
WL204	WL203, metabolically evolved in xylose with improved anaerobic growth	This study
Plasmid		
pKD4	FRT-*kan*-FRT cassette	[16]
pKD46	*bla*, red recombinase, temperature-dependent replication	[16]
pFT-A	*bla*, *flp*, temperature-dependent replication	[20]
Primers		
Clone *ldhL*-P1	CCTATTATTTATGGCGGTGTCGTTT	This study
Clone *ldhL*-P2	CAGTTCGCTGACTGTAAGTTGTTGC	This study
Delete *adhE*-P1	ATGGCTGTTACTAATGTCGCTGAACTTAACGCAC	This study
	TCGTAGAGC**GTGTGTAGGCTGGAGATGCTTC**^1)^	
Delete *adhE*-P2	TTAAGCGGATTTTTTCGCTTTTTTCTCAGCTTTAG	This study
	CCGGAGC**AGCCATATGAATATCCTCCTTAG**^1)^	
Verify △*adhE*-P1	TGATGAAGGCTAATGCTG	This study
Verify △*adhE*-P2	CTTACGCCACCTGGAAGT	This study
Verify insertion *ldhL*-P1	GGTTCTAGTTACGCATTCG	This study
Verify insertion *ldhL*-P2	CTTCTTCTTTTCGTCATCG	This study

1. The bold sequence is homologous to the flanked sequence of the FRT-kan-FRT cassette in pKD4.

### Genetic methods

Standard methods were used for DNA transformation, electroporation, PCR amplification, and analyses of DNA fragments. Chromosomal gene deletion and integration was carried out using previously described λ red homologous recombination procedures [10,17]. The gene deletions and integrations were verified by using appropriate antibiotic markers and analysis of PCR and fermentation products.

### Fermentations

Seed cultures were prepared by inoculating fresh colonies from LB-xylose plates into 500 ml flasks containing 200 ml LB medium with 2% (w/v) xylose, and incubated for 10 h (37°C, 150 rpm) until they achieved an OD_600nm_ of ~1.9 (0.665 g L^-1^ cell dry weight). Seed cultures were inoculated (with a starting OD_600nm_ of 0.1) into a 7-L fermenter (Sartorius Stedim Biotech GmbH 37070, Germany) containing 4-L LB medium with 70 g L^-1^ xylose. The fermentation was carried out for 96 h (37°C, 200 rpm, and pH 7). The pH was controlled by automatic addition of 6 N KOH. Samples (1.5 ml) were taken periodically for analysis of cell growth, sugar consumption and lactic acid production. Data presented were the averages of three replicated fermentations.

### Analysis

Cell growth was estimated from the optical density (1-L cells with an OD_600nm_ of 1 is equivalent to 0.35 g dry cell weight). Fermentation samples were centrifuged at 8,000 rpm for 10 min. The supernatant was then filtered through a 0.22 μm membrane, and used for HPLC analysis of sugar and organic acids concentrations (BioRad HPX 87H column, 35°C, 0.5 ml min^-1^ of 4 mM H_2_SO_4_ as the mobile phase). Optical purity was determined by HPLC using a chiral column (EC 250/4 NUCLEOSIL Chiral-1, Germany) (35°C, 0.5 ml min^-1^ of 0.2 mM CuSO_4_ as the mobile phase) and D (-) and L (+)-lactic acids (Sigma-Aldrich) as the standards.

## Competing interests

The authors declare that they have no competing interests.

## Authors’ contribution

JZ, LZ and YW carried out the genetic modification; XZ and JW participated the fermentation; EG, RM and SZ summarized the data and wrote the manuscript. All authors read and approved the final manuscript.
